# “Cruising together”—ASC specks and SAA, a perfect match in chronic inflammation

**DOI:** 10.1038/s44321-024-00109-y

**Published:** 2024-07-30

**Authors:** Salie Maasewerd, Bernardo Simoes Franklin

**Affiliations:** Institute of Innate Immunity, Medical Faculty, University of Bonn, Bonn, Germany

**Keywords:** Immunology

## Abstract

S. Maasewerd and B. Franklin discuss a recent study by Aguzzi and colleagues (in this issue of EMBO Mol Med) that uncovers the important role played by the inflammasome adaptor ASC in the pathogenesis of AA amyloidosis.

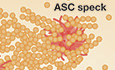

## Teaser

Currently, over 350 million people worldwide live with chronic inflammatory diseases, facing a range of complications that severely impact their quality of life. Among these complications is amyloidosis, a group of diseases characterized by the extracellular deposition of insoluble amyloid fibrils that disrupt tissue structure and function. One specific form, amyloid A (AA) amyloidosis, is closely linked to chronic inflammatory conditions such as rheumatoid arthritis (RA), Familial Mediterranean Fever (FMF), Crohn’s disease, and chronic infections. In this issue of *EMBO Mol Med*, Losa et al, shows that the inflammasome adaptor ASC plays an important role in the pathogenesis of AA amyloidosis, and suggests a potential immunotherapy for treating the disease (Losa et al, 2024).

AA amyloidosis results from tissue deposition of serum amyloid A (SAA), a soluble acute-phase protein produced by the liver during inflammation. SAA levels skyrocket during inflammatory responses reaching up to 1000-fold of their basal levels. If not rapidly cleared, SAA can accumulate as amyloid deposits in tissues and organs, primarily in the kidneys, liver, and spleen. Typically, spleen macrophages clear SAA via Fc-receptor-mediated phagocytosis. However, Losa and colleagues provide new evidence that macrophages may be the same cells that contribute to AA amyloid deposition in the first place.

Upon encountering with various insults (such as pathogens and stressors), macrophages assemble inflammasomes. These intracellular molecular machineries process the maturation and release of pro-inflammatory cytokines of the interleukin 1 (IL-1) family. Among those, IL-1β is closely related to AA amyloidosis as SAA levels rapidly decline in patients undergoing IL-1β-inhibition (ClinicalTrials.gov, NCT00069329). However, novel evidence, including the new study from Losa and colleagues, support an IL-1-independent contribution of inflammasomes to AA amyloidosis. Once activated, inflammasomes trigger the oligomerization of the adaptor protein ASC (apoptosis-associated speck-like protein containing a CARD), forming the so-called ASC specks. These specks play a vital role in the pro-inflammatory response by maximizing IL-1 maturation, and facilitating pyroptosis, an inflammatory type of cell death characterized by plasma membrane rupture and the leakage of intracellular content from the cell.

Notably, even before inflammasomes were identified as primary genetic causes, amyloidosis was a known hallmark of the so-called autoinflammatory syndromes diseases, which today are known to be caused by mutations in genes coding for inflammasome proteins. Patients often present with numerous long-term complications, including glomerulonephritis, with AA amyloid deposits in the glomeruli, tubules, and interstitium of the kidneys.

Already in 2008, Banu Balci-Peynircioglu and colleagues discovered that ASC specks are present in amyloid deposits in the kidneys of FMF patients, suggesting a potential role for ASC in amyloid formation (Balci-Peynircioglu et al, 2008). The authors posited two hypotheses: (1) ASC specks from dying cells may persist in the extracellular space, and (2) extracellular ASC specks (eASC) might nucleate amyloid. It took seven more years for the first hypothesis to be proven correct (Baroja-Mazo et al, 2014; Franklin et al, 2014).

Apart from its well-established role as an inflammasome adaptor, ASC has extracellular functions that persist beyond pyroptosis. eASC outlive the cells that produced them, sustaining IL-1 inflammation by cleaving these cytokines in the extracellular space or by activating inflammasomes in cells that engulf eASC (Baroja-Mazo et al, 2014; Franklin et al, 2014). Additional studies have confirmed the formation and release of ASC specks in vivo during infections, chronic inflammatory diseases, and demonstrated their accumulation in tissues of humans and mice (Baroja-Mazo et al, 2014; Gaul et al, 2021; Tzeng et al, 2016; Basiorka et al, 2018; Venegas et al, 2017). Furthermore, eASC has pathological and IL-1-independent relevance in gout and rheumatoid arthritis (Bertheloot et al, 2022).

**Figure 1:**
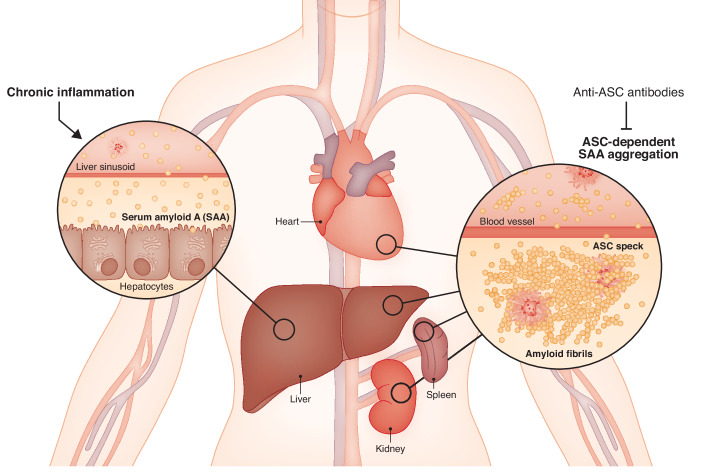
ASC specks cross-seed serum amyloid A (SAA) aggregates. During inflammation, liver hepatocytes secrete the acute phase protein serum amyloid A (SAA) into the circulation. SAA levels can reach 1000-fold their basal levels and if not rapidly cleared can accumulate in tissues, including the liver, heart, spleen, and kidneys. Inflammation also induces the activation of inflammasomes and the release of ASC specks from dying inflamed cells. In chronic inflammation, extracellular ASC specks (eASC) form and accumulate in the liver (de Carvalho Ribeiro et al, 2023; Gaul et al, 2021), spleens (Tzeng et al, 2016), kidneys (Balci-Peynircioglu et al, 2008) and in the circulation (Baroja-Mazo et al, 2014; Basiorka et al, 2018), suggesting numerous sites where these two proteins could meet. In this study, Losa and co-workers show that eASC interact with SAA via its Pyrin domain, forming a scafold that accellerates SAA aggregation into amyloid fibrils, which deposit in tissues. The study provides compelling evidence for the IL-1-independent role for eASC in chronic inflammation.

The second hypothesis gained strength in 2017, with the discovery that eASC accelerated amyloid-β deposition in Alzheimer’s disease (Venegas et al, 2017), another amyloid precursor that shares large similarities to AA. Still, even though ASC specks may be tied to the development of AA amyloidosis, it remained to be shown whether eASC actively affect amyloid deposition during this process. Now, another 10 years from the discovery of eASC, Losa and co-workers provided compelling evidence that eASC can nucleate SAA aggregation (Fig. 1). The authors found that ASC forms complexes with SAA in tissue of a patient with AA amyloidosis. In a series of elegant experiments, Losa and colleagues demonstrated that ASC specks significantly contribute to the aggregation and deposition of SAA in AA amyloidosis. Using super resolution microscopy, they showed that ASC tightly colocalizes with SAA in human amyloid deposits, suggesting a direct interaction. Mass spectrometry revealed that this interaction occurs through the pyrin domain of ASC, which the authors confirmed accelerates SAA fibrillation in vitro. Using murine models of amyloidosis, they found that mice lacking ASC (*Pycard*^−/−^) had significantly reduced amyloid deposits compared to wild-type controls. Moreover, targeting ASC with anti-ASC antibodies significantly reduced amyloid deposition in vivo in these models (Fig. 1), identifying ASC as a viable therapeutic strategy for AA amyloidosis.

Notably, recent studies demonstrated that eASC accumulates and persist in the liver, the main site of SAA production (de Carvalho Ribeiro et al, 2023; Gaul et al, 2021). In mouse models of chronic liver diseases, eASC accumulation in the liver perpetuates inflammation and damage. Hence, the findings of Losa and co-workers offer an enticing area of investigations and contribute to the mounting evidence of inflammasome-independent roles for ASC, as demonstrated in diseases like RA and multiple sclerosis, in which *Pycard*^−/−^ mice exhibit more severe phenotypes than mice lacking *Nlrp3* or *IL-1*.

This research profoundly impacts our understanding of amyloid diseases and opens novel therapeutic interventions. By demonstrating another example of ASC’s prion-like behavior in amyloid deposition, Losa’s work provides critical insights into the mechanisms of AA amyloidosis and highlights the potential of targeting ASC for therapeutic purposes, an area of growing interest for small biotech and big pharma (Coll et al, 2022). This research not only expands our understanding of the extracellular functions of inflammasomes but also offers promising new avenues for the treatment of chronic inflammatory and amyloid diseases. As the field progresses, it will be crucial to further explore the therapeutic potential of anti-ASC strategies and to understand the broader implications of ASC specks in various diseases.
